# Amyloid-β Oligomer-Induced Electrophysiological Mechanisms and Electrical Impedance Changes in Neurons

**DOI:** 10.3390/s24041211

**Published:** 2024-02-14

**Authors:** Shimeng Sun, Qing Ma, Qiyu Sheng, Shangwei Huang, Chenxia Wu, Junsong Liu, Jia Xu

**Affiliations:** 1 Department of Physiology and Pharmacology, Health Science Center, Ningbo University, Ningbo 315211, China; 2111101023@nbu.edu.cn (S.S.); maqing@nbu.edu.cn (Q.M.); 196002075@nbu.edu.cn (Q.S.); hswhty@163.com (S.H.); 18368618533@163.com (C.W.); 2 State Key Laboratory of Superhard Materials, College of Physics, Jilin University, Changchun 130012, China

**Keywords:** Aβ oligomer, Alzheimer’s disease, electrical impedance spectrum, CPE-equivalent electrical circuit model, cytotoxicity

## Abstract

Amyloid plays a critical role in the pathogenesis of Alzheimer’s disease (AD) and can aggregate to form oligomers and fibrils in the brain. There is increasing evidence that highly toxic amyloid-β oligomers (AβOs) lead to tau protein aggregation, hyperphosphorylation, neuroinflammation, neuronal loss, synaptic loss, and dysfunction. Although the effects of AβOs on neurons have been investigated using conventional biochemical experiments, there are no established criteria for electrical evaluation. To this end, we explored electrophysiological changes in mouse hippocampal neurons (HT22) following exposure to AβOs and/or naringenin (Nar, a flavonoid compound) using electrical impedance spectroscopy (EIS). AβO-induced HT22 showed a decreased impedance amplitude and increased phase angle, and the addition of Nar reversed these changes. The characteristic frequency was markedly increased with AβO exposure, which was also reversed by Nar. The AβOs decreased intranuclear and cytoplasmic resistance and increased nucleus resistance and extracellular capacitance. Overall, the innovative construction of the eight-element CPE-equivalent circuit model further reflects that the pseudo-capacitance of the cell membrane and cell nucleus was increased in the AβO-induced group. This study conclusively revealed that AβOs induce cytotoxic effects by disrupting the resistance characteristics of unit membranes. The results further support that EIS is an effective technique for evaluating AβO-induced neuronal damage and microscopic electrical distinctions in the sub-microscopic structure of reactive cells.

## 1. Introduction

Alzheimer’s disease (AD) is a common degenerative disease that affects the nervous system. Given the aging population worldwide, AD has emerged as a pressing health issue in recent years. The pathology of AD is characterized by the presence of senile plaques formed by amyloid deposits and neurofibrillary tangles formed by large tau protein aggregates. Soluble amyloid-β oligomers (AβOs) are widely recognized as the trigger of neuronal damage that leads to AD [1]. Amyloid-β is a small polypeptide produced by the sequential cleavage of the amyloid precursor protein caused by β-secretase and γ-secretase that is capable of aggregating to form monomers (Aβ monomers, AβMs), oligomers (Aβ oligomers, AβOs), and fibrils (Aβ fibrils, AβFs) [2]. While all Aβ aggregates are neurotoxic, oligomers are particularly toxic and can induce a cascade of reactions that ultimately lead to the clinical symptoms of AD [3]. The different conformations of Aβ in AD may contribute to its pathology via distinct mechanisms. For example, AβOs impair synaptic plasticity and memory [4,5]; significantly disrupt synaptic composition, morphology, and density [6]; disrupt synaptic activity [7]; lead to resulting synapse loss [8]; accelerate specific neuronal cell death [9]; and inhibit long-term potentiation (LTP) [4]. The activation of primary astrocytes by Aβ results in the phosphorylation of tau proteins in neurons [10], triggers oxidative stress [11], and induces neuroinflammation due to the release of pro-inflammatory factors such as TNFα and IL-1β [12].

Numerous studies have highlighted the cognitive benefits associated with consuming flavonoid-rich foods in individuals with normal cognitive function. Naringenin (Nar), a natural flavonoid abundant in citrus fruits, has garnered attention because of its diverse biological activities, including anti-inflammatory, antioxidant, and anti-cancer properties [13]. Research by Ghofrani S et al. highlighted the potential of Nar to mitigate Aβ-induced impairment in learning and memory [14]. The neuroprotective effects of Nar arise from its ability to inhibit microglial activation, reduce the expression of inflammatory mediators, and reduce neuronal cell death [15]. To further explore these neuroprotective effects, this study used electrical assessment to evaluate the cytotoxic effects of Aβ oligomers employing Nar as a protective agent.

Given the significance of Aβ proteins in AD pathology, they have attracted attention for identifying clinical biomarkers, investigating pathogenic mechanisms, monitoring AD manifestation, and developing drug therapies. However, conventional methods in AD research are subject to various limitations. As cognitive test results can be impacted by a patient’s mood and external factors, they are employed for initial clinical screening purposes. Imaging techniques, such as magnetic resonance imaging (MRI) and Aβ positron emission tomography (PET) imaging, can provide visual insights into pathological brain changes and are considered the gold standard for AD clinical diagnosis. However, these medical imaging procedures, which are expensive and require specialized equipment, are most suitable for the examination of mid-to-late-stage AD patients [16]. Alternatively, the early monitoring of AD progression to enable timely intervention can be achieved by detecting Aβ biomarkers in cerebrospinal (CSF) fluid. However, CSF sampling is a highly invasive procedure with low patient compliance [17]. Blood sampling is not suitable because the concentration of Aβ in plasma is significantly lower than in CSF because of the filtering effect of the blood–brain barrier. Moreover, the results are susceptible to interference from metabolic factors, indirectly reflecting the brain’s Aβ metabolism levels [18]. Given the neurotoxic effects of Aβ proteins, it is imperative to develop novel methods to rapidly, sensitively, and precisely quantify Aβ. Such advancements would be of significant value for AD screening, diagnosis, and treatment.

Electrical impedance spectroscopy (EIS) is a non-invasive, label-free electrokinetic technique for the precise characterization of cellular electrical properties [19]. EIS is currently used in diverse fields, including materials science [20], electrochemistry [21], and bioelectronics [22,23], among others [24]. EIS can analyze a wide range of sample types, including tissues, cells [25], and proteins [26]. Given these features, EIS is instrumental for the real-time, dynamic profiling of electrical features in both normal and pathological cells and can provide profound insights into their ever-changing dynamics and responses to pharmacological or toxic stimuli [27]. This method hinges upon cell polarization induced by an electric field and the intricate interplay of ions at the cellular membrane [28]. EIS harnesses a sophisticated blend of mathematical, physical, and circuit-modeling techniques that enable the precise extraction of critical information pertaining to cellular impedance, capacitance, and resistance [29,30,31], as well as morphological parameters and physiological status, such as type [32], concentration [33], and viability [34]. To date, impedance-based biosensors have shown extensive applications for discriminating diverse cell types from humans and other species, harnessing the nuanced dielectric properties encompassing impedance and capacitance [35,36,37]. However, few studies have dynamically and plastically monitored neurotoxic protein-induced neuronal cell damage from an electrical perspective. Furthermore, there are limited supporting data and references for electrical parameter indices and foundations that can be used to achieve drug improvement or protection against neuronal cell damage. The establishment of real-time monitoring techniques for neuronal cell phenotype changes is thus of paramount importance for passive electrophysiological research into understanding the pathogenic mechanisms of AD. 

In this study, we utilized EIS technology to quantify the impedance behavior of individual cells using a Cole–Cole mathematical model and an innovative eight-element-CPE–resistor–capacitor equivalent circuit model to obtain the characteristic phase angle, amplitude, and frequency measurements. We then compared the effects of normal conditions, Aβ-induced neurotoxicity, and plant-derived neuroprotective molecule Nar on impedance, capacitance, and resistance changes in a mouse hippocampal neuronal cell line (HT22). By doing so, this study provides an electrical perspective and data support for unraveling the mechanisms of neuronal cell damage and developing novel AD treatment strategies.

## 2. Materials and Methods

### 2.1. Materials and AβO Preparation

Synthetic Aβ_1-42_ was purchased from GL Biochem (Shanghai, China). The Aβ_1-42_ oligomer utilized in this study was prepared following a meticulously optimized protocol based on prior work [38]. In brief, Aβ_1-42_ was dissolved in an appropriate quantity of 1,1,1,3,3,3-Hexafluoro-2-propanol (HFIP, Aladdin Biochemical Technology Co., Ltd., Shanghai, China) and then allowed to stand at room temperature for 20 min. Subsequently, an appropriate amount of ddH_2_O was added and left at room temperature for an additional 20 min. The solution was then centrifuged at 14,000 rpm for 15 min, and the resulting supernatant was collected. The HFIP was volatilized by nitrogen blowing. The Aβ solution was obtained after continuous shaking at room temperature for 48 h. AβM and AβF were prepared for comparative verification of dot blot experiments, as previously described [39].

### 2.2. Transmission Electron Microscopy (TEM)

AβO solution (10 μL) was added dropwise to the copper mesh and allowed to dry. Samples were stained three times with 2% phosphotungstic acid, and excess staining solution was removed via suction using clean filter paper. Lastly, the copper mesh was thoroughly examined using a TEM (Hitachi, Tokyo, Japan).

### 2.3. Particle Size Measurements

After the samples were prepared as described above, they were diluted and added to the cuvette. The measurement temperature was set to 25 °C, and the optical path was set to 1 cm; other test parameters were set according to the Malvern Zetasizer Nano series instructions (Malvern, UK). The reported measurement result is the average of three scanning results.

### 2.4. Dot-Blotting Analysis

Samples (2 μL) were evenly spaced on a polyvinylidene fluoride (PVDF) membrane and allowed to dry naturally. Subsequently, the membrane was blocked for 2 h in TBST solution containing 5% milk and then incubated for 1 h with anti-oligomer antibody A11 (Thermo Fisher Scientific, Waltham, MA, USA, AHB0052, 1:1000) or anti-Aβ antibody B4 (Santa Cruz Biotechnology, Dallas, TX, USA, sc-28365, 1:1000) with mild shaking. After washing three times with TBST, the membranes were incubated with secondary antibodies for 1 h. Lastly, the protein was visualized using ECL Western blotting detection reagents (NCM Biotech, Suzhou, China).

### 2.5. Cell Culture

The mouse hippocampal neuronal cell line (HT22) was procured from Fuheng Biotechnology Co., LTD. (Shanghai, China) and cultured in high-glucose modified Eagle’s medium (DMEM) supplemented with 10% fetal bovine serum (FBS), 100 U/mL penicillin, and 100 μg/mL streptomycin. Cells were seeded into a 75 cm^2^ cell culture flask and maintained in an incubator containing 5% CO_2_ at 37 °C. The medium was replaced every other day to ensure cell viability. HT22 cells were pre-treated with AβO (HT22 + AβO) for 24 h in the AD model group. HT22 cells were pre-treated with AβO followed by exposure to Nar (≥95%, Sigma-Aldrich Co. LLC, Shanghai, China) for 24 h (HT22 + AβO + Nar) in the medication group. The control group (HT22) was not exposed to AβO or Nar. Before AC electrical impedance measurements, HT22 cells from each group were harvested with trypsin and then resuspended to achieve a consistent cell density.

### 2.6. Cell Viability Assessment

The optimal concentration of AβO to establish a neurotoxic-protein-induced AD model was determined based on prior studies [40,41]. We determined the optimal therapeutic concentration of Nar under AβO induction conditions through cell viability experiments. In brief, HT22 cells were plated on a 96-well plate and incubated at 37 °C with 5% CO_2_ for 24 h. When the HT22 cells attained an appropriate density, AβO and Nar were sequentially added (except for the control group). The following day, 10 μL of MTT (3-(4,5-Dimethylthiazol-2-yl)-2.5-diphenyltetrazolium bromide) was added to each well. After 4 h of incubation, the supernatant was discarded, and 100 μL of dimethyl sulfoxide (DMSO) was added to each well. Cell viability was assessed as the absorbance at 490 nm using a Varioskan LUX Multimode Microplate Reader (Thermo Fisher Scientific Inc.).

### 2.7. Impedance Spectroscopy Measurements

**Figure 1:**
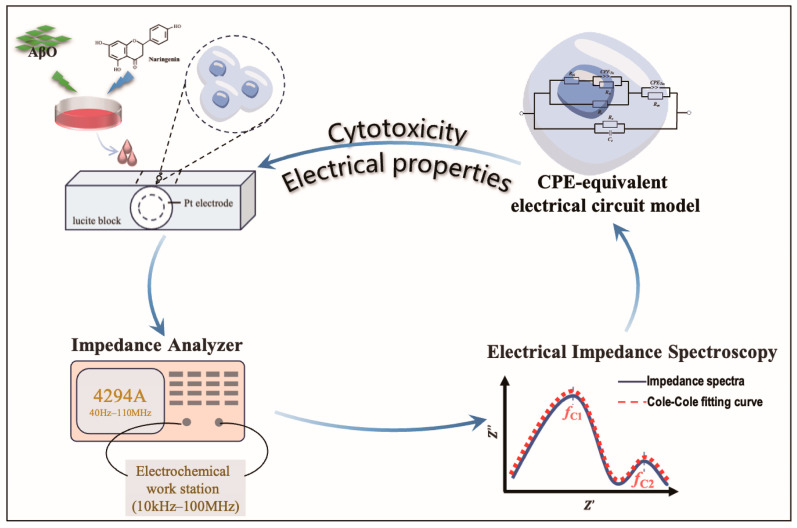
Diagram of impedance spectroscopy measurements.

HT22 cells, including adherent and post-drug-induced suspended cells under different induction conditions, were collected in 15 mL centrifuge tubes. Enriched cell samples were obtained via centrifugation at 1000 rpm for 5 min. For impedance measurement, the cells were suspended in a quartz sample chamber featuring parallel double platinum electrodes, following previously described specifications [25]. The amplitude, |Z|, and phase angle, *θ*, of the HT22 cells were assessed using an Agilent 4294A impedance analyzer (Agilent Technologies, Santa Clara, CA, USA) equipped with an Agilent 42942A terminal adapter and an Agilent 16192A parallel electrode SMD test fixture. Dispersion characteristics were measured in a frequency range of 10 kHz to 100 MHz with 124 frequency points at room temperature (Figure 1).

To minimize the impact of electrode polarization, impedance data were normalized. DMEM culture medium without fetal bovine serum (FBS) and penicillin/streptomycin (P/S) was used as a detecting buffer for cell suspension. The impedance value of the detecting buffer was normalized. The cell impedance ($Z=Z_{cell}$) is the difference between the measured impedance ($Z_{measure}$) and the extracellular fluid impedance ($Z_{medium}$). The complex impedance was expressed as $Z_{measure}=\left|Z_{measure}\right|\times e^{-j\theta }=Z_{measure}^{\prime }+jZ_{measure}^{\prime \prime }$. The real ($Z_{measure}^{\prime }$) and imaginary ($Z_{measure}^{\prime \prime }$) impedances were calculated as $Z_{measure}^{\prime }=\left|Z_{measure}\right|\times \cos\theta$ and $Z_{measure}^{\prime \prime }=\left|Z_{measure}\right|\times \sin\theta$, respectively. Each run was measured three times each. The average of these three runs was used as the data point, i.e., ${Z=Z_{cell}=Z}_{measure}-Z_{polarization}$, $Z_{polarization}=Z_{medium}$.

### 2.8. Parameter Analysis of Cole-Cole Mathematical Model

In HT22 mouse hippocampal neurons, the presence of a cell nucleus gives rise to two β-dispersions at the interface of the cell membrane and nuclear membrane. In this context, we employed a comprehensive 9-parameter dual-dispersion Cole–Cole model for impedance spectroscopy as follows:

(1) $$Z\left(s\right)=R_{\infty }+\frac{∆R_{1}}{1+{\left({jf/f}_{c1}\right)}^{\beta_{1}}}+\frac{{∆R}_{2}}{1+{\left({jf/f}_{c2}\right)}^{\beta_{2}}}$$

$R_{\infty }$ is the extra-high-frequency resistance to accommodate the non-zero value of the impedance at high frequencies. ∆R_1_, ∆R_2_; j; f; *f*_C1_, *f*_C2_; and β_1_, β_2_ represent the relaxation increments at low- and high-frequency, the imaginary unit ($j=\sqrt{-1}$), the frequency, the characteristic frequencies, and the Cole–Cole parameters (0 < β < 1), respectively [42].

These parameters were calculated from the real ($Z^{\prime }$) and imaginary ($Z^{\prime \prime }$) impedance to experimental values and the real ($Z_{theory}^{\prime }$) and imaginary ($Z_{theory}^{\prime \prime }$) impedance to the theoretical values with the Cole–Cole model using the equations below to minimize the residual, where ∆ denotes the difference between the maximum and the minimum number of relevant observed values within the frequency range considered, and n is the number of frequency points covered by that range [43]:

(2) $$Res\left(Z^{\prime }\right)=\frac{\sum \left|Z^{\prime }-Z_{theory}^{\prime }\right|}{∆Z^{\prime }}\times \frac{100}{n}$$

(3) $$Res\left(Z^{\prime \prime }\right)=\frac{\sum \left|Z^{\prime \prime }-Z_{theory}^{\prime \prime }\right|}{∆Z^{\prime \prime }}\times \frac{100}{n}$$

### 2.9. Eight-Element CPE-Equivalent Circuit Model

**Figure 2:**
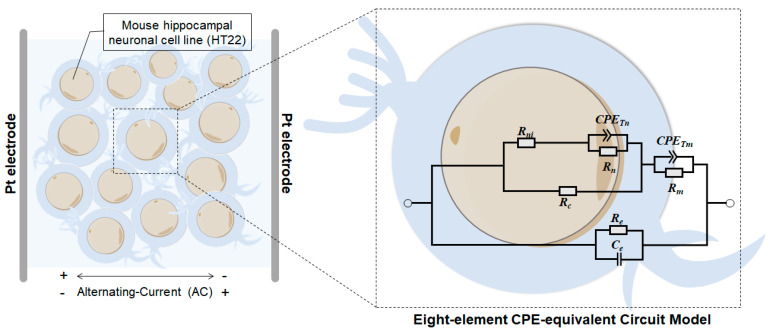
Schematic of the eight-element CPE-equivalent circuit model for neurons.

Mouse hippocampal neurons may not conform to the “ideal” capacitor model assumed in standard electrical elements [30]. In our experiments, the constant phase element (CPE) model (Figure 2) proved to be the most accurate fit for the experimental data [44]. Within this model, the cell membrane and nuclear membrane possess their own resistance ($R_{m}$ and $R_{n}$, respectively) and pseudo-capacitance (${CPE}_{Tm}$, ${CPE}_{Tn}$). In contrast, the intranuclear and cytoplasmic components exhibit purely resistive behavior ($R_{ni}$, $R_{c}$). The equivalent parallel resistance ($C_{e}$) and capacitance ($R_{e}$) circuit represent the capacitance of the extracellular fluid. Notably, the equivalent circuit model reflects the state of cell suspensions under assumed conditions, where the structural electrical characteristics (cell membrane, cytoplasm, cell nucleus) of all cells in the test sample are identical. A suspension comprising several identical cells (the sample) can represent the average state of an individual cell, which is assumed to be completely identical. The eight-element CPE-equivalent circuit model constructed in this study is essentially a circuit model of these assumed fully identical individual cells. Using a fitting analysis of this circuit model, the true variations in the measured data curves can be more accurately reflected. The use of this model to characterize the electrical properties of homogenous individual cells under assumed conditions is reasonable. Considering the parallel and serial combinations of the elements within the eight-element CPE-equivalent circuit mode, impedance can be expressed as follows.

(4) $$Z=\left.\left(C_{e}\left\|Z_{e}\right.\right)\right\|\left\{\left\{\left.\left[\left(Z_{{CPE}_{n}}\left\|Z_{n}\right.\right)+Z_{ni}\right]\right\|Z_{c}\right\}+\left(Z_{{CPE}_{m}}\left\|Z_{m}\right.\right)\right\}$$

where $Z_{k}=R_{k}$ for $\kappa \in \left(e,m,n,ni,c\right)$; $Z_{k}\left(s\right)={\left(S^{\alpha_{l}}\cdot {CPE}_{T_{l}}\right)}^{-1}$ for $l\in \left(m,n\right)$; and the dispersion coefficient $\alpha ={CPE}_{p}$. Hence, the impedance within the equivalent circuit model, as applied through fractional circuit theory [45], can be expressed as

(5) $$Z\left(s\right)={\left\{{\left(sC_{e}+\frac{1}{R_{e}}\right)}^{-1}+{\left[{\left(\frac{1}{\left(S^{\alpha_{n}}\right)\cdot CPE_{Tn}+\frac{1}{R_{n}}}+\frac{1}{R_{ni}}\right)}^{-1}+\frac{1}{R_{c}}\right]}^{-1}+\frac{1}{\left(S^{\alpha_{m}}\right)\cdot CPE_{Tm}+\frac{1}{R_{m}}}\right\}}^{-1}$$

where s=jω. The complex impedance of (5) can be calculated using the replacement $s^{\alpha_{i}}=\omega^{\alpha_{i}}\left[\cos\left(\frac{\pi \cdot \alpha_{i}}{2}\right)+j\sin\left(\frac{\pi \cdot \alpha_{i}}{2}\right)\right]$. The parameters were acquired by applying an automated curve-fitting process to HT22 impedance spectrum data using the Zview2 3.1 Software.

### 2.10. Statistical Analysis

Data are reported as the mean ± standard deviation (SD). Differences between the control (HT22), AβO-induced (HT22 + AβO), and Nar with AβO-induced (HT22 + AβO + Nar) groups were assessed using Student’s *t*-test. Statistical analysis was performed using the SPSS 12.0 software (IBM, Armonk, NY, USA). Results with a *p*-value < 0.05 were considered statistically significant. 

## 3. Results

### 3.1. Characterization of AβOs

**Figure 3:**
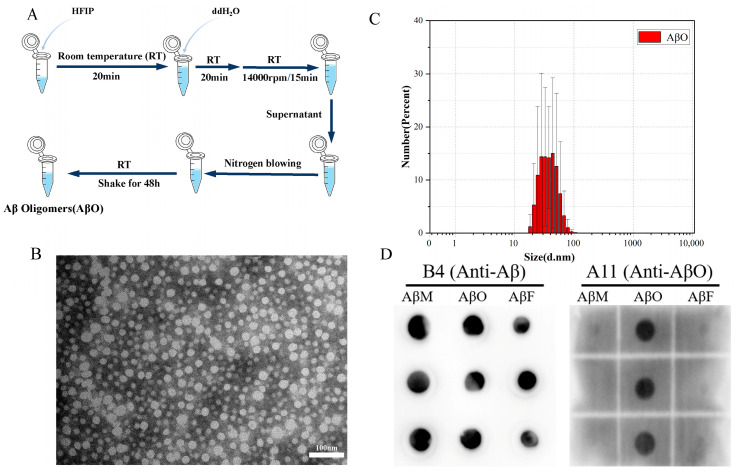
Characterization of AβOs. (**A**) Protocol for AβO preparation. (**B**) TEM images of AβOs (scale bar = 100 nm). (**C**) Particle size distribution by DLS. (**D**) Dot-blotting analysis of AβMs, AβOs, and AβFs using an anti-oligomer antibody (A11) and a general anti-Aβ antibody (B4). Three parallel experiments were performed as replications.

AβOs were prepared and characterized as shown in Figure 3A. Clear and uniformly distributed oligomers were observed in the AβO samples using TEM (Figure 3B), suggesting that AβOs are uniformly distributed spherical particles with a diameter of approximately 60 nm. The average particle size of the protein solution was roughly distributed between 20 and 80 nm via dynamic light scattering (DLS) (Figure 3C). Anti-oligomer antibody A11 does not recognize monomers or mature fibers and reacts only with oligomers. To further verify the accuracy and specificity of the preparation of the toxic neuroprotein AβOs, we also prepared AβMs and AβFs as previously described [39] for comparison. The dot-blotting assay indicated the accurate preparation of the homogeneous, stable, and specific neurotoxic protein AβOs (Figure 3D).

### 3.2. Effects of AβOs and Nar on HT22 Cell Impedance Spectra

To model AD and evaluate the neuroprotective effects of Nar, we first conducted a cell viability assay to optimize the AβO induction conditions. The results (Figure S1) indicated that 1.0 μM of AβO was significantly neurotoxic (*p* < 0.001). Nar significantly improved AβO-induced neuronal damage in a dose-dependent manner within a certain range. Nar exerted protective effects against the induction of Aβ oligomers at doses of both 50 and 100 μM, consistent with prior reports [46,47]. Considering the optimal protective effect at 50 μM Nar and the need to conserve reagents, we used this concentration for subsequent experiments.

**Figure 4:**
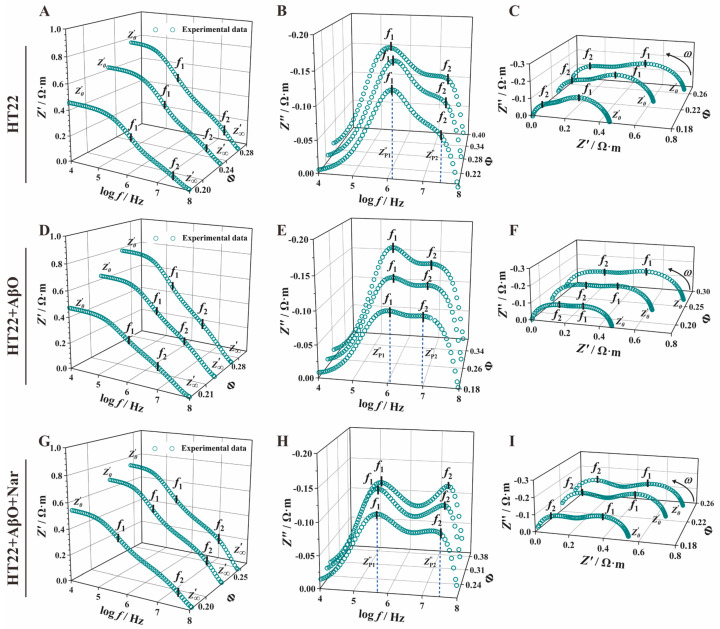
Impedance spectra of the control (HT22), AβO-induced (HT22 + AβO), and Nar with AβO-induced (HT22 + AβO + Nar) groups. (**A**,**D**,**G**) The real part of the impedance–frequency spectrum ($Z^{\prime }-f$). (**B**,**E**,**H**) The imaginary part of the impedance–frequency spectrum ($Z^{\prime \prime }-f$). (**C**,**F**,**I**) Nyquist plots ($Z^{\prime }-Z^{\prime \prime }$). Green dots indicate the experimental data.

To ensure consistent HT22 cell concentrations, the concentration was adjusted within a range of approximately 22–23%, with no significant differences between groups. Figure 4 shows a clear representation of the impedance spectra curves for HT22, HT22 + AβO, and HT22 + AβO + Nar, demonstrating similar overall trends with noticeable differences in characteristic peaks. Figure 4A,D,G display the real part of the impedance–frequency spectrum, [$Z^{\prime }\left(f\right)$curve]. The limits of the impedance ($Z_{0}^{\prime }$) reflect the electrical properties of the extracellular fluid. The HT22 cell suspension exhibited a high impedance characteristic of capacitance at low frequencies (10 kHz–100 kHz, log⁡f= 4–5). As the frequency was increased from 0.1 MHz to 10 MHz (log⁡f= 5–7), the capacitive reactance of the cell membrane and HT22 cell suspension decreased because of the incomplete polarization of the cells. This resulted in distinctive peak and valley features in the impedance spectrum, with characteristic frequencies ($f_{1}$ and $f_{2}$) corresponding to the high impedance characteristics of the cell membrane and nuclear membrane. At high electric field frequencies (>10 MHz, log⁡f= 7), there is insufficient time for the cells to polarize. Consequently, the current flows sequentially through the extracellular fluid, cell membrane, cytoplasm, nuclear membrane, and nuclear contents sequentially, resulting in distinct peaks and valleys in the impedance spectrum. Importantly, the $Z^{\prime }\left(f\right)$ curve continues as $Z^{\prime }$ decreases to $Z_{\infty }^{\prime }$, which performs the capacitive short circuit of the membrane. The limit of the real part of the impedance ($Z_{\infty }^{\prime }$) represents the electrical characteristics of the nuclear contents. After exposure to AβO, the $Z^{\prime }\left(f\right)$ curve of the exposed group shifted to the low-impedance region, manifesting slight changes in $Z_{0}^{\prime }$ and significant improvement in $Z_{\infty }^{\prime }$, with a notable recovery observed in the Nar-protected group (HT22 + AβO + Nar). 

The imaginary part of the impedance ($Z^{\prime \prime }$) showed a double-peak sheer semicircular curve (Figure 4B,E,H). At 10 kHz, after normalization to eliminate the influence of electrode polarization, the $Z^{\prime \prime }$ value of HT22 was close to zero, exhibiting a steep upward trend with an increasing frequency. In a range of 0.1 to 1 MHz (log⁡f= 5–6), a single characteristic peak formed at the cell membrane interface, which was followed by cytoplasmic polarization loss due to the induced charges. Subsequently, a prominent feature peak reflected the polarization phenomenon of the nuclear membrane around 10 MHz. This was followed by a rapid decrease in Z″, indicating the electrical loss of nuclear content and the presentation of a distinctive “double-peak sandwiched by a single valley” spectrum. The characteristic peak has four parameters: the first and second peaks of the imaginary part of impedance ($Z_{P1}^{\prime \prime }$, $Z_{P2}^{\prime \prime }$) and the first and second characteristic frequencies ($f_{1},\;f_{2}$). Notably, each group exhibited distinct peak–valley features. AβO exposure increased the double peaks compared with the HT22 control group, while Nar induction resulted in significant recovery compared with the AβO-exposed group.

Nyquist plots for electrical impedance spectroscopy displayed a double-peak semicircular curve with an oblate shape (Figure 4C,F,I), indicating significant variation in characteristic peaks among the groups. The centers of the double semicircles were located below the abscissa, and their graphical definitions in terms of vertices and height represented ($f_{1},\;Z_{P1}^{\prime \prime }$) and ($f_{2},\;Z_{P2}^{\prime \prime }$), respectively. The control group (HT22 cells) exhibited a characteristic pattern with high peaks at low frequencies and low peaks at high frequencies. The observations were similar in the AβO-induced group (HT22 + AβO). In the Nar group (HT22 + AβO + Nar), the first and second characteristic peaks were relatively evenly distributed with minor differences. 

### 3.3. Impedance Spectrum Parameter Comparative Analysis

To enable a more in-depth comparison of the characteristic parameters, we performed impedance parameter extraction (Table 1). The comparisons revealed no significant difference in the impedance amplitude at a low frequency ($Z_{0}^{\prime }\;$= |Z|_0_). However, the high-frequency limit of the real part of impedance ($Z_{\infty }^{\prime }$) in the AβO-induced group ($Z_{\infty ,A\beta O}^{\prime }$ = (0.24 ± 0.23) × 10^−2^ Ω·m) was significantly reduced compared with the HT22 group ($Z_{\infty ,HT22}^{\prime }$ = (1.12 ± 0.28) × 10^−2^ Ω·m), with a decrease of 78.57%, whereas the Nar-induced group showed a significant recovery to ($Z_{\infty ,Nar}^{\prime }$ = (0.71 ± 0.2) × 10^−2^ Ω·m). This phenomenon applies to a frequency range extending from several kilohertz to several megahertz, which is commonly referred to as β-dispersion [48]. Unexpectedly, the addition of AβOs slightly decreased the imaginary part of impedance ($Z_{P2}^{\prime \prime }$) compared with the HT22 group ($Z_{P2,A\beta O}^{\prime \prime }$ = −0.13 ± 0.02 Ω·m, $Z_{P,Nar}^{\prime \prime }$ = −0.10 ± 0.02 Ω·m). Compared with the HT22 group ($\theta_{P,HT22}$ = −30.27 ± 1.3 deg), the AβO group ($\theta_{P,A\beta O}$ = −31.53 ± 0.59 deg) showed an increased phase angle, while the presence of Nar ($\theta_{P,Nar}$ = −25.4 ± 1.25 deg) reversed this change. A significant decrease was also observed in the characteristic frequency ($f_{2,\;HT22}$ = 28.55 ± 0.87 MHz) of the AβO group ($f_{2,\;A\beta O}$ = 9.99 ± 0.80 MHz), while there was recovery in the Nar group ($f_{2,\;Nar}$ = 28.11 ± 6.04 MHz). The Nar treatment significantly recovered the impedance characteristics of the cells and nuclear membranes induced by AβO neurotoxicity.

### 3.4. Extraction of Mathematical Model Parameters

**Figure 5:**
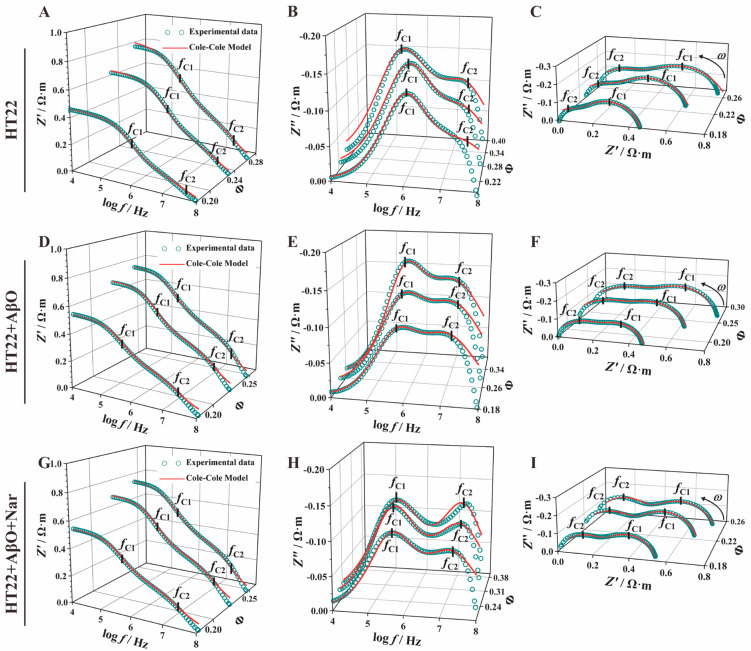
Impedance spectra and Cole–Cole fitting curve of the control (HT22), AβO-induced (HT22 + AβO), and Nar for the AβO-induced (HT22 + AβO + Nar) groups. (**A**,**D**,**G**) The real part of the impedance–frequency spectrum ($Z^{\prime }-f$). (**B**,**E**,**H**) The imaginary part of the impedance–frequency spectrum ($Z^{\prime \prime }-f$). (**C**,**F**,**I**) Nyquist plots ($Z^{\prime }-Z^{\prime \prime }$). Green dots and red lines indicate the experimental data and the Cole–Cole mathematical model fit curve, respectively.

To further examine the electrical characteristics of HT22 cells under AβO and Nar conditions, we employed the Cole–Cole equation for mathematical fitting and subsequent parameter extraction from the impedance spectra. Figure 5 illustrates the Cole–Cole equation fit curves (depicted by the red lines). The results exhibit remarkable alignment with the measured impedance spectrum data, indicating that the Cole–Cole mathematical model and extracted parameters reflect the true characteristics of the curve and changes, highlighting the accuracy of the Cole–Cole equation in extracting impedance spectrum information. The extracted parameters are presented in Table 2. The AβO group ($f_{C1,\;A\beta O}$ = (6.01 ± 0.27) × 10^5^ Hz) exhibited a significantly lower $f_{C1}$ than the control group ($f_{C1,\;HT22\;}$= (8.25 ± 2.62) × 10^5^ Hz) and Nar ($f_{C1,\;Nar}$ = (3.77 ± 0.27) × 10^5^ Hz). Conversely, the second characteristic frequency ($f_{C2}$) of the AβO group ($f_{C2,\;A\beta O}$ = (2.03 ± 0.18) × 10^7^ Hz) was lower than that of the control group ($f_{C2,\;HT22}$ = (4.00 ± 0.99) × 10^7^ Hz) but was reversed by the addition of Nar ($f_{C2,\;Nar}$ = (2.88 ± 0.28) × 10^7^ Hz). The first characteristic frequency reflects changes in the cell membrane, while the second relates to changes in the nucleus. The characteristic frequency is a signature parameter reflecting cellular responses to alternating electromagnetic fields and is thus a significant indicator of cellular electrophysiological properties in physiologically important frequency domains. Notably, the addition of AβOs led to a slight reduction in the first Cole–Cole parameter and a marked decrease in the second Cole–Cole parameter.

### 3.5. Fitting an Equivalent Electrical Circuit Model with Constant Phase Element 

**Figure 6:**
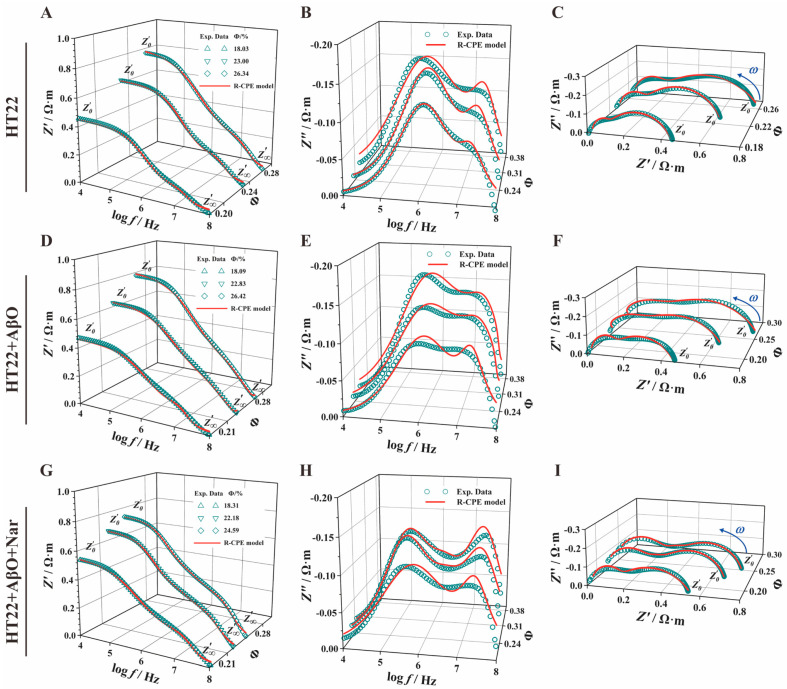
Effect of AβOs and Nar on equivalent circuit parameters of HT22 cells. A 3D stereogram of the real part of impedance–concentration (**A**,**D**,**G**), the imaginary part of the impedance–concentration (**B**,**E**,**H**), and a Nyquist plot (**C**,**F**,**I**). $R_{ni}$ represents intranuclear resistance, ${CPE}_{Tn}$ represents the constant phase element of the cell nucleus, $R_{n}$ represents nuclear envelope resistance, $R_{c}$ represents cytoplasmic resistance, ${CPE}_{Tm}$ represents the constant phase element of the cell membrane, $R_{m}$ represents cell membrane resistance, $R_{e}$ represents extracellular resistance, and $C_{e}$ represents extracellular capacitance. The green hollow data points and red solid lines indicate the experimental data and CPE-equivalent electrical circuit model fitting results, respectively.

Treating an electrochemical system as a circuit system comprising various electronic and ionic components allows us to describe the system’s electrochemical properties using the electrical properties of the circuit components. By fitting an equivalent circuit model to EIS data, relevant electrochemical information can be extracted from the spectra. In this way, we obtained different concentrations of HT22 cell impedance spectra to clarify the electrical parameters of nerve cells. To do so, we designed a novel eight-element CPE-equivalent circuit model (where $R_{ni}$ represents intranuclear resistance, ${CPE}_{Tn}$ represents the constant phase element of the cell nucleus, $R_{n}$ represents nucleus resistance, $R_{c}$ represents cytoplasmic resistance, ${CPE}_{Tm}$ represents the constant phase element of the cell membrane, Rm represents cell membrane resistance, $R_{e}$ represents extracellular resistance, and $C_{e}$ represents extracellular capacitance) to accurately extract circuit model parameters in the subsequent steps. As depicted in Figure 6, the three groups showed a similar trend with increasing frequency. The green hollow point lines represent the measurement data of cells at various concentrations, while the red solid lines depict the fitting results of the CPE-equivalent circuit model. By fitting the measured data and circuit model, we can accurately extract characteristic parameters and obtain electrical parameter information related to the cell membrane, cytoplasm, and other subcellular structures.

### 3.6. Extraction of Electrical Circuit Model Parameters

The cell structural parameters (Table 3) are presented based on the eight-element CPE-equivalent electrical circuit model (Figure 6) and obtained via the automatic curve fitting of microglia impedance spectrum data using the Zview2 3.1 Software. Exposure to AβOs increased the extracellular capacitance ($R_{e,A\beta O}$ = 0.64 ± 0.10 Ω·m) and cytoplasmic resistance ($R_{c,A\beta O}$, = 0.43 ± 0.20 Ω·m); the rates of change were 0.46% and 0.04% compared with the HT22 group, respectively. Compared with the HT22 group, a magnitude difference in change was observed compared with the HT22 group in cell membrane resistance ($R_{m,A\beta O}$ = (5.84 ± 1.31) × 10^5^ Ω·m, *p* < 0.01), nuclear membrane resistance ($R_{n,A\beta O}$, = 3.79 ± 2.94 Ω·m, *p* < 0.01), and intranuclear resistance ($R_{ni,A\beta O}$, = 0.41 ± 0.20 Ω·m, *p* < 0.01). 

For the Nar group, the cell circuit model parameters ($C_{e}$, $R_{e}$, $R_{m}$, $R_{c}$, $R_{n}$, and $R_{ni}$) closely resembled those of the HT22 group, indicating varying degrees of improvement and protection under Nar-induced conditions compared with AβO-induced toxic damage induced by AβOs. Significant group differences were also observed in the pseudo-capacitance and dispersion coefficients of the cell membrane (${CPE}_{Tm}$, $\alpha_{m}$) and nuclear membrane (${CPE}_{Tn}$, $\alpha_{n}$). With the addition of protection, these parameters exhibited significant reversal.

## 4. Discussion

There is growing evidence that AβO is a crucial biomarker of neurotoxic damage, inflammatory responses, synaptic loss, and excessive phosphorylation of tau proteins in AD [3]. Excessive AβO accumulation in the cortex and hippocampal regions triggers a cascade of pathological manifestations, including neurodegeneration, brain atrophy, and cognitive impairment [49]. AβO induction plays a pivotal role in AD pathogenesis by triggering neuronal oxidative stress and synaptic damage, ultimately resulting in neuronal dysfunction, atrophy, and cell loss [50]. At present, it is challenging to track neurotoxic protein-induced neuronal cell damage in real-time from an electrical standpoint, and there are insufficient data to establish electrical parameter benchmarks and groundwork for enhancing drug interventions or protecting against neuronal cell damage. 

Impedance spectroscopy enables the detection of the resistance and capacitance responses of materials in different frequency ranges under alternating electric fields and variable temperatures. By employing mathematical modeling and circuit analysis, this method can provide valuable insights into the internal structural characteristics of substances that exceed the ability of conventional experimental approaches, effectively affording a unique “sample perspective”. Several studies have applied EIS in this way. Li et al. [51] conducted a quantitative analysis of red blood cell aggregability (AG) in coagulated blood within an extracorporeal circulation system using impedance spectroscopy combined with the modified Hanai formula. Adopting a mathematical–physics modeling perspective of cellular electrical properties, Sabuncu et al. [52] employed EIS to acquire the dielectric properties of isolated neuroendocrine adrenal chromaffin cells utilizing Cole–Cole and Maxwell–Wagner mixture models for fitting. The determined parameters included the specific capacitance of chromaffin cells and secretory granule membranes (1.22 and 7.10 μF/cm^2^, respectively), cytoplasmic conductivity (excluding and including the influence of intracellular membranous structures, 1.14 and 0.49 S/m, respectively), and secretory granule milieu conductivity (0.35 S/m). To assess a cell’s physiological state, Şimşek et al. applied EIS to analyze the impact of GO and rGO on electrical measurements and human carotid endothelial cell (HCtAEC) proliferation [53]. Notably, Tran et al. established a non-invasive, rapid, real-time method for detecting red blood cell (RBC) hemolysis based on EIS and developed an eight-parameter equivalent circuit to characterize electrode polarization, the typical circuit of blood, cytoplasm, and interfacial polarization portions [54]. To study compound intervention in cellular-stress-induced pathological changes, Yin et al. applied impedance to study drug responses [55]. In a previous study, we utilized EIS, including Bode plots, Nyquist plots, and Nichols plots, to analyze blood samples from mice exposed to lead. The results revealed a significant reduction in impedance and the phase angle characteristics of red blood cells and an increase in the first and second characteristic frequencies [30].

In the present study, we investigated the impedance characteristics of the mouse hippocampal neuronal cell line HT22 using EIS. The extracted impedance spectrum parameters determined the real part ($Z_{\infty }^{\prime }$) and amplitude (${\left|Z\right|}_{\infty }$) of impedance, the phase angle ($\theta_{P}$), and the characteristic frequency ($f_{1}$, $f_{2}$), providing objective data on the impedance characteristics of HT22 cells under healthy conditions. These findings will help address the current lack of electrical characteristic parameters for neuronal cells and provide meaningful references for the impedance properties of neuronal cells, including but not limited to astrocytes, microglia, oligodendrocytes, and other types of neurons. Furthermore, we utilized the Cole–Cole model to elucidate the mathematical parameters of HT22 cells under AβO-induced conditions. The characteristic frequency ($f_{C1}$, $f_{C2}$) and impedance increment (${\Delta R}_{1}$, ${\Delta R}_{2}$) established a correlation between cellular structure and mathematical model parameters, indicating that AβO-induced membrane damage in HT22 cells indirectly affects the integrity of the cell nucleus. 

To further evaluate the value of EIS for drug screening, we monitored the electrical effects of Nar on nerve cells. Nar is a natural flavonoid commonly found in citrus fruits that are known to have various biological activities, including anti-inflammatory, antioxidant, and anti-cancer properties [56]. Nar shows preventive and therapeutic effects for several neurological diseases, such as AD and Parkinson’s disease [57]. Although several equivalent electrical circuit models have been established [58], we extended this approach by optimizing the traditional single-mode capacitive-resistive equivalent circuit model. Drawing inspiration from the CPE, we designed and constructed an innovative eight-element CPE-equivalent electrical circuit model that is more precise and suitable for applications involving neural cells. 

Through the analysis of HT22 cell sub-microstructures using the CPE-equivalent electrical circuit model, we demonstrated that exposure to AβOs led to a nearly one-order-of-magnitude reduction in cell membrane resistance ($R_{m}$). After Nar protection, the recovery of AβO-induced cell membrane resistance damage approached levels similar to those of normal cells. Regarding cellular nuclear protective mechanisms under stress conditions, we observed significant differences in cell nucleus resistance ($R_{n}$) under AβO-induced toxicity and Nar protection conditions, with these differences also exhibiting a noticeable reversal trend. Similar reversal phenomena were evident in the pseudo-capacitance and dispersion coefficients of the cell membrane (${CPE}_{Tm}$, $\alpha_{m}$) and nuclear membrane (${CPE}_{Tn}$, $\alpha_{n}$), further emphasizing that the neurotoxic effects of AβO and the neuroprotective actions of Nar can be accurately expressed through precise fitting using cellular circuit models. Together, these results indicate that AβO-induced damage to the sub-microstructures of HT22 cells is manifested by an increase in cell membrane capacitance and resistance. Nar was shown to significantly ameliorate neuro-membrane and electrophysiological dysfunction caused by AβOs, demonstrating its neuroprotective potential. These findings align with previous physiological and biochemical studies demonstrating AβO-induced neurotoxic damage and the neuroprotective effects of Nar [46,59]. Furthermore, the residual values from the CPE-equivalent electrical circuit model fitting were in the order of 10^−2^, indicating an excellent fit between the circuit model designed in this study and the measured impedance curves, supporting the accuracy and precision of cell sub-microstructure electrical parameter extraction.

In this study, we utilized an Agilent 4294A impedance analyzer to acquire impedance spectra from mouse hippocampal neurons. These spectra were then analyzed using both the Cole–Cole mathematical model and a CPE-equivalent electrical circuit model. Through this electrical approach, we evaluated the detrimental effects of AβOs and the protective effects of Nar on mouse hippocampal neurons. The precise extraction of impedance parameters, mathematical model parameters, and equivalent circuit model parameters has enabled us to develop a groundbreaking method of electrical assessment and detecting AβO-induced damage in neuronal cells. Furthermore, we established a comprehensive set of parametric indicators designed for use in impedance spectroscopy screening systems. We believe that the assessment of nerve cell electrophysiology represents a key direction for future neurobiological EIS applications. This approach offers immense promise for advancing our understanding of disease mechanisms and facilitating drug development. However, it should be noted that our investigation was limited to the comprehensive electrical assessment of neuronal cell suspensions. Briefly, we used an equivalent circuit model to reflect the state of the cell suspension under the assumed conditions and that the structural electrical properties of all cells in the tested samples are identical. Indeed, a suspension consisting of several identical cells can precisely reflect the same average state of a single cell. Nevertheless, this work has established a robust foundation by providing dependable neuronal data parameters and highly accurate solutions for fitting equivalent circuit models, setting the stage for obtaining passive electrophysiological profiles of single cells in the near future. We firmly believe that the non-invasive electrical characterization of neurons and other neural cells based on impedance spectroscopy is an urgent topic in biomedical research with significant applications for thorough explorations of the brain.

## 5. Conclusions

This study evaluated the toxic effects of AβOs and the protective effects of Nar on mouse hippocampal neurons through the detection of impedance spectra and the resolution of the Cole–Cole mathematical model and a CPE-equivalent electrical circuit model. The results showed that AβOs decreased the real part of impedance, impedance amplitude, intranuclear, and cytoplasmic resistance and increased the phase angle, characteristic frequency, nucleus resistance, extracellular capacitance, and pseudo-capacitance of the cell membrane and cell nucleus. Notably, the addition of Nar reversed these changes. These findings show that EIS is a suitable method for evaluating neuronal injury and revealing microscopic electrical differences in the sub-microscopic structure of reactive cells induced by AβO. Data on differences in the electrical properties of neuronal cells under Aβ oligomer intervention compared with non-intervention controls using a dielectric assay will be of value for the future development of novel Aβ-related AD biomarkers.

## Figures and Tables

**Table 1:** Impact of AβO and Nar on electrical impedance spectra in HT22 Cells.

Parameters	Symbol/Unit	HT22	HT22 + AβO	HT22 + AβO + Nar
Low-frequency limit of real part of impedance	$Z_{0}^{\prime }$/Ω·m	0.62 ± 0.10	0.62±0.10	0.64±0.07
High-frequency limit of real part of impedance	$Z_{\infty }^{\prime }$/Ω·m	(1.12 ± 0.28) × 10^−2^	(0.24 ± 0.23) × 10^−2^ ***	(0.71 ± 0.2) × 10^−2 ###^
Real-part increment of electrical impedance	∆*Z*/Ω·m	0.61 ± 0.09	0.62 ± 0.09	0.63 ± 0.07
Impedance amplitude at high frequency	|*Z*|_∞_/Ω·m	0.03 ± 0.01	0.03 ± 0.01	0.04 ± 0.02 ^#^
Impedance amplitude increment	∆|*Z*|/Ω·m	0.60 ± 0.08	0.60 ± 0.08	0.59 ± 0.05
The 1st peak of imaginary part of impedance	$Z_{P1}^{\prime \prime }$/Ω·m	−0.16 ± 0.01	−0.14 ± 0.02	−0.13 ± 0.01
The 2nd peak of imaginary part of impedance	$Z_{P2}^{\prime \prime }$/Ω·m	−0.10 ± 0.02	−0.13 ± 0.02 **	−0.12 ± 0.02
Peak of phase angle (deg)	*θ*_P_/deg	−30.27 ± 1.3	−31.53 ± 0.59 *	−25.4 ± 1.25 ^###^
The 1st characteristic frequency	*f*_1_/MHz	1.05 ± 0.26	0.94 ± 0.11	0.45 ± 0.10 ^###^
The 2nd characteristic frequency	*f*_2_/MHz	28.55 ± 0.87	9.99 ± 0.80 ***	28.11 ± 6.04 ^###^

Note: Impedance amplitude at low frequency ($Z_{0}^{\prime }$ = |Z|_0_). Real-part increment of electrical impedance ($∆Z=Z_{0}^{\prime }-Z_{\infty }^{\prime }$). Impedance amplitude increment ($∆\left|Z\right|={\left|Z\right|}_{0}-{\left|Z\right|}_{\infty }$). Significance is indicated as follows: * for HT22 vs. HT22 + AβO; ^#^ for HT22 + AβO vs. HT22 + AβO + Nar: *^/#^ *p* < 0.05, ** *p* < 0.01, ***^/###^ *p* < 0.001. Ten concentration repetitions per group (n = 10).

**Table 2:** Mathematical model parameters of HT22 cells induced by AβOs and Nar using the Cole–Cole equation.

Parameters	Symbol/Unit	HT22	HT22 + AβO	HT22 + AβO + Nar
Cell volume fraction	Փ	0.23 ± 0.03	0.23 ± 0.03	0.22 ± 0.02
The limiting impedance at high frequency	R_∞/_Ω·m	−0.04 ± 0.00	−0.05 ± 0.01 **	−0.03 ± 0.02 ^#^
The first characteristic frequency	*f*_C1_/Hz	(8.25 ± 2.62) × 10^5^	(6.01 ± 0.27) × 10^5^ *	(3.77 ± 0.27) × 10^5 ###^
The first impedance increment	∆R_1/_Ω·m	0.43 ± 0.06	0.3 ± 0.05 ***	0.34 ± 0.05
The first Cole–Cole parameter	*β*_1_	0.74 ± 0.04	0.8 ± 0.04 **	0.78 ± 0.02
The second characteristic frequency	*f*_C2_/Hz	(4 ± 0.99) × 10^7^	(2.03 ± 0.18) × 10^7^ ***	(2.88 ± 0.28) × 10^7 ###^
The second impedance increment	∆R_2/_Ω·m	0.26 ± 0.06	0.39 ± 0.08 ***	0.34 ± 0.03
The second Cole–Cole parameter	*β*_2_	0.71 ± 0.03	0.66 ± 0.03 **	0.72 ± 0.06 ^#^
Residual	Res(Z′)/%	1.24 ± 0.18	1.27 ± 0.30	1.16 ± 0.35
	Res(Z″)/%	4.69 ± 0.39	4.41 ± 1.18	3.81 ± 0.86

Significance is indicated as follows: * for HT22 vs. HT22 + AβO; ^#^ for HT22 + AβO vs. HT22 + AβO + Nar: *^/#^ *p* < 0.05, ** *p* < 0.01, ***^/###^ *p* < 0.001. Ten concentration repetitions per group (n = 10).

**Table 3:** Electric component values of the established equivalent circuit model.

Parameters	Symbol/Unit	HT22	HT22 + AβO	HT22 + AβO + Nar
Extracellular capacitance	$C_{e}$	(1.19 ± 2.58) × 10^−9^	(2.19 ± 0.7) × 10^−8^ ***	(1.63 ± 0.45) × 10^−8 #^
Extracellular resistance	$R_{e}$/Ω·m	0.63 ± 0.10	0.64 ± 0.10	0.71 ± 0.18
Pseudo-capacitance of cell membrane	${CPE}_{Tm}$/$nF\cdot s^{\alpha_{m}-1}$	(1.54 ± 1.78) × 10^−5^	(2.89 ± 0.7) × 10^−5^ *	(0.95 ± 1.56) × 10^−5 ##^
Dispersion coefficient of cell membrane	$\alpha_{m}$	0.74 ± 0.05	0.67 ± 0.02 **	0.81 ± 0.06 ^###^
Cell membrane resistance	$R_{m}$/Ω·m	(4.48 ± 2.63) × 10^6^	(5.84 ± 1.31) × 10^5^ **	(2.11 ± 0.89) × 10^6 ###^
Pseudo-capacitance of cell nucleus	${CPE}_{Tn}$/$nF\cdot s^{\alpha_{n}-1}$	(5.45 ± 7.09) × 10^−9^	(0.36 ± 1.15) × 10^−10^ *	(2.64 ± 4.08) × 10^−7^
Dispersion coefficient of cell nucleus	$\alpha_{n}$	1.16 ± 0.08	1.79 ± 0.19 ***	1.00 ± 0.12 ^###^
Nuclear envelope resistance	$R_{n}$/Ω·m	0.31 ± 0.14	3.79 ± 2.94 **	0.94 ± 0.79 ^#^
Intranuclear resistance	$R_{ni}$/Ω·m	0.03 ± 0.01	0.41 ± 0.20 ***	0.65 ± 0.40
Cytoplasmic resistance	$R_{c}$/Ω·m	0.43 ± 0.08	0.43 ± 0.20	1.09 ± 0.40 ^###^
Chi-sqr	χ^2^	(2.47 ± 1.65) × 10^−2^	(2.66 ± 1.61) × 10^−2^	(1.27 ± 0.62) × 10^−2 #^
Sum of sqr	∑χ^2^	5.91 ± 3.93	6.36 ± 3.85	3.04 ± 1.48 ^#^

Significance is indicated as follows: * for HT22 vs. HT22 + AβO; ^#^ for HT22 + AβO vs. HT22 + AβO + Nar: *^/#^ *p* < 0.05, **^/##^ *p* < 0.01, ***^/###^ *p* < 0.001.

## Data Availability

The data will be made available upon request.

## Supplementary Materials

The following supporting information can be downloaded at: https://www.mdpi.com/article/10.3390/s24041211/s1, Figure S1: Cellular viability of AβOs and Nar on HT22 cells.

## References

[1] Wang X., Kastanenka K.V., Arbel-Ornath M., Commins C., Kuzuya A., Lariviere A.J., Krafft G.A., Hefti F., Jerecic J., Bacskai B.J. (2018). An acute functional screen identifies an effective antibody targeting amyloid-β oligomers based on calcium imaging. Sci. Rep..

[2] Selkoe D.J. (2001). Alzheimer’s disease: Genes, proteins, and therapy. Physiol. Rev..

[3] Serrano-Pozo A., Frosch M.P., Masliah E., Hyman B.T. (2011). Neuropathological alterations in Alzheimer disease. Cold Spring Harb. Perspect. Med..

[4] Shankar G.M., Li S., Mehta T.H., Garcia-Munoz A., Shepardson N.E., Smith I., Brett F.M., Farrell M.A., Rowan M.J., Lemere C.A. (2008). Amyloid-beta protein dimers isolated directly from Alzheimer’s brains impair synaptic plasticity and memory. Nat. Med..

[5] Bao F., Wicklund L., Lacor P.N., Klein W.L., Nordberg A., Marutle A. (2012). Different β-amyloid oligomer assemblies in Alzheimer brains correlate with age of disease onset and impaired cholinergic activity. Neurobiol. Aging.

[6] Lacor P.N., Buniel M.C., Furlow P.W., Clemente A.S., Velasco P.T., Wood M., Viola K.L., Klein W.L. (2007). Abeta oligomer-induced aberrations in synapse composition, shape, and density provide a molecular basis for loss of connectivity in Alzheimer’s disease. J. Neurosci..

[7] Wang Z., Jackson R.J., Hong W., Taylor W.M., Corbett G.T., Moreno A., Liu W., Li S., Frosch M.P., Slutsky I. (2017). Human Brain-Derived Aβ Oligomers Bind to Synapses and Disrupt Synaptic Activity in a Manner That Requires APP. J. Neurosci..

[8] Deshpande A., Mina E., Glabe C., Busciglio J. (2006). Different conformations of amyloid beta induce neurotoxicity by distinct mechanisms in human cortical neurons. J. Neurosci..

[9] Viola K.L., Klein W.L. (2015). Amyloid β oligomers in Alzheimer’s disease pathogenesis, treatment, and diagnosis. Acta Neuropathol..

[10] Watanabe K., Uemura K., Asada M., Maesako M., Akiyama H., Shimohama S., Takahashi R., Kinoshita A. (2015). The participation of insulin-like growth factor-binding protein 3 released by astrocytes in the pathology of Alzheimer’s disease. Mol. Brain.

[11] Nguyen C.D., Yoo J., Hwang S.Y., Cho S.Y., Kim M., Jang H., No K.O., Shin J.C., Kim J.H., Lee G. (2022). Bee Venom Activates the Nrf2/HO-1 and TrkB/CREB/BDNF Pathways in Neuronal Cell Responses against Oxidative Stress Induced by Aβ(1-42). Int. J. Mol. Sci..

[12] Jian M., Kwan J.S., Bunting M., Ng R.C., Chan K.H. (2019). Adiponectin suppresses amyloid-β oligomer (AβO)-induced inflammatory response of microglia via AdipoR1-AMPK-NF-κB signaling pathway. J. Neuroinflamm..

[13] Kannappan S., Palanisamy N., Anuradha C.V. (2010). Suppression of hepatic oxidative events and regulation of eNOS expression in the liver by naringenin in fructose-administered rats. Eur. J. Pharmacol..

[14] Ghofrani S., Joghataei M.T., Mohseni S., Baluchnejadmojarad T., Bagheri M., Khamse S., Roghani M. (2015). Naringenin improves learning and memory in an Alzheimer’s disease rat model: Insights into the underlying mechanisms. Eur. J. Pharmacol..

[15] Wu L.H., Lin C., Lin H.Y., Liu Y.S., Wu C.Y., Tsai C.F., Chang P.C., Yeh W.L., Lu D.Y. (2016). Naringenin Suppresses Neuroinflammatory Responses Through Inducing Suppressor of Cytokine Signaling 3 Expression. Mol. Neurobiol..

[16] Scheltens P., De Strooper B., Kivipelto M., Holstege H., Chételat G., Teunissen C.E., Cummings J., van der Flier W.M. (2021). Alzheimer’s disease. Lancet.

[17] Jack C.R., Knopman D.S., Jagust W.J., Shaw L.M., Aisen P.S., Weiner M.W., Petersen R.C., Trojanowski J.Q. (2010). Hypothetical model of dynamic biomarkers of the Alzheimer’s pathological cascade. Lancet Neurol..

[18] Blennow K., Zetterberg H. (2018). Biomarkers for Alzheimer’s disease: Current status and prospects for the future. J. Intern. Med..

[19] Crowell L.L., Yakisich J.S., Aufderheide B., Adams T.N.G. (2020). Electrical Impedance Spectroscopy for Monitoring Chemoresistance of Cancer Cells. Micromachines.

[20] Huang R., Carr C.G., Gopal C.B., Haile S.M. (2022). Broad Applicability of Electrochemical Impedance Spectroscopy to the Measurement of Oxygen Nonstoichiometry in Mixed Ion and Electron Conductors. ACS Appl. Mater. Interfaces.

[21] Strong M.E., Richards J.R., Torres M., Beck C.M., La Belle J.T. (2021). Faradaic electrochemical impedance spectroscopy for enhanced analyte detection in diagnostics. Biosens. Bioelectron..

[22] Wang L., Hu S., Liu K., Chen B., Wu H., Jia J., Yao J. (2020). A hybrid Genetic Algorithm and Levenberg-Marquardt (GA-LM) method for cell suspension measurement with electrical impedance spectroscopy. Rev. Sci. Instrum..

[23] Turcan I., Caras I., Schreiner T.G., Tucureanu C., Salageanu A., Vasile V., Avram M., Tincu B., Olariu M.A. (2021). Dielectrophoretic and Electrical Impedance Differentiation of Cancerous Cells Based on Biophysical Phenotype. Biosensors.

[24] Heileman K., Daoud J., Tabrizian M. (2013). Dielectric spectroscopy as a viable biosensing tool for cell and tissue characterization and analysis. Biosens. Bioelectron..

[25] Schwarz M., Jendrusch M., Constantinou I. (2020). Spatially resolved electrical impedance methods for cell and particle characterization. Electrophoresis.

[26] Choi Y., Hong S., Kang T., Lee L.P. (2009). Noninvasive Real-time Monitoring of Amyloid-β Fibrillization via Simultaneous Label-free Dielectric Relaxation Spectroscopy and Dark-Field Imaging. J. Phys. Chem. C.

[27] Eker B., Meissner R., Bertsch A., Mehta K., Renaud P. (2013). Label-free recognition of drug resistance via impedimetric screening of breast cancer cells. PLoS ONE.

[28] Spencer D., Morgan H. (2020). High-Speed Single-Cell Dielectric Spectroscopy. ACS Sens..

[29] Xu J., Xie W., Chen Y., Wang L., Ma Q. (2020). Dielectric properties of nucleated erythrocytes as simulated by the double spherical-shell model. Chin. Physics B.

[30] Yang B., Xu J., Hu S., You B., Ma Q. (2021). Effects of lead exposure on blood electrical impedance spectroscopy of mice. Biomed. Eng. Online.

[31] Xu J., Cen L., Ma Q. (2022). Evaluating Membrane Electrical Properties of SMMC7721 Cells with TiO_2_ NPs Applications to Cytotoxicity by Dielectric Spectroscopy. J. Biomed. Nanotechnol..

[32] Gawad S., Schild L., Renaud P.H. (2001). Micromachined impedance spectroscopy flow cytometer for cell analysis and particle sizing. Lab Chip.

[33] Sabounchi P., Morales A.M., Ponce P., Lee L.P., Simmons B.A., Davalos R.V. (2008). Sample concentration and impedance detection on a microfluidic polymer chip. Biomed. Microdevices.

[34] David F., Hebeisen M., Schade G., Franco-Lara E., Di Berardino M. (2012). Viability and membrane potential analysis of Bacillus megaterium cells by impedance flow cytometry. Biotechnol. Bioeng..

[35] Giana F.E., Bonetto F.J., Bellotti M.I. (2018). Assay based on electrical impedance spectroscopy to discriminate between normal and cancerous mammalian cells. Phys. Rev. E.

[36] Li J., Wan N., Wen J., Cheng G., He L., Cheng L. (2019). Quantitative detection and evaluation of thrombus formation based on electrical impedance spectroscopy. Biosens. Bioelectron..

[37] Kadan-Jamal K., Sophocleous M., Jog A., Desagani D., Teig-Sussholz O., Georgiou J., Avni A., Shacham-Diamand Y. (2020). Electrical Impedance Spectroscopy of plant cells in aqueous biological buffer solutions and their modelling using a unified electrical equivalent circuit over a wide frequency range: 4Hz to 20 GHz. Biosens. Bioelectron..

[38] Chunhui H., Dilin X., Ke Z., Jieyi S., Sicheng Y., Dapeng W., Qinwen W., Wei C. (2018). A11-positive β-amyloid Oligomer Preparation and Assessment Using Dot Blotting Analysis. J. Vis. Exp..

[39] Sulatsky M.I., Sulatskaya A.I., Povarova O.I., Antifeeva I.A., Kuznetsova I.M., Turoverov K.K. (2020). Effect of the fluorescent probes ThT and ANS on the mature amyloid fibrils. Prion.

[40] Xiang S., Liu F., Lin J., Chen H., Huang C., Chen L., Zhou Y., Ye L., Zhang K., Jin J. (2017). Fucoxanthin Inhibits β-Amyloid Assembly and Attenuates β-Amyloid Oligomer-Induced Cognitive Impairments. J. Agric. Food Chem..

[41] Wang J., Zheng J., Huang C., Zhao J., Lin J., Zhou X., Naman C.B., Wang N., Gerwick W.H., Wang Q. (2018). Eckmaxol, a Phlorotannin Extracted from *Ecklonia maxima*, Produces Anti-β-amyloid Oligomer Neuroprotective Effects Possibly via Directly Acting on Glycogen Synthase Kinase 3β. ACS Chem. Neurosci..

[42] Elwakil A.S., Al-Ali A.A., Maundy B.J. (2021). Extending the double-dispersion Cole-Cole, Cole-Davidson and Havriliak-Negami electrochemical impedance spectroscopy models. Eur. Biophys. J..

[43] Watanabe M., Suzaki T., Irimajiri A. (1991). Dielectric behavior of the frog lens in the 100 Hz to 500 MHz range. Simulation with an allocated ellipsoidal-shells model. Biophys. J..

[44] Grimnes S., Martinsen O.G. (2005). Cole electrical impedance model—A critique and an alternative. IEEE Trans. Biomed. Eng..

[45] Herencsar N., Freeborn T.J., Kartci A., Cicekoglu O. (2020). A Comparative Study of Two Fractional-Order Equivalent Electrical Circuits for Modeling the Electrical Impedance of Dental Tissues. Entropy.

[46] Zhang N., Hu Z., Zhang Z., Liu G., Wang Y., Ren Y., Wu X., Geng F. (2018). Protective Role Of Naringenin Against Aβ(25-35)-Caused Damage via ER and PI3K/Akt-Mediated Pathways. Cell Mol. Neurobiol..

[47] Heo H.J., Kim D.O., Shin S.C., Kim M.J., Kim B.G., Shin D.H. (2004). Effect of antioxidant flavanone, naringenin, from *Citrus junoson* neuroprotection. J. Agric. Food Chem..

[48] Markx G.H., Davey C.L. (1999). The dielectric properties of biological cells at radiofrequencies: Applications in biotechnology. Enzym. Microb. Technol..

[49] Frost P.S., Barros-Aragão F., da Silva R.T., Venancio A., Matias I., Lyra E.S.N.M., Kincheski G.C., Pimentel-Coelho P.M., De Felice F.G., Gomes F.C.A. (2019). Neonatal infection leads to increased susceptibility to Aβ oligomer-induced brain inflammation, synapse loss and cognitive impairment in mice. Cell Death Dis..

[50] Yu H., Yamashita T., Hu X., Bian Z., Hu X., Feng T., Tadokoro K., Morihara R., Abe K. (2022). Protective and anti-oxidative effects of curcumin and resveratrol on Aβ-oligomer-induced damage in the SH-SY5Y cell line. J. Neurol. Sci..

[51] Li J., Sapkota A., Kikuchi D., Sakota D., Maruyama O., Takei M. (2018). Red blood cells aggregability measurement of coagulating blood in extracorporeal circulation system with multiple-frequency electrical impedance spectroscopy. Biosens. Bioelectron..

[52] Sabuncu A.C., Stacey M., Craviso G.L., Semenova N., Vernier P.T., Leblanc N., Chatterjee I., Zaklit J. (2018). Dielectric properties of isolated adrenal chromaffin cells determined by microfluidic impedance spectroscopy. Bioelectrochemistry.

[53] Şimşek F., Can O.M., Garipcan B., Kocatürk Ö., Ülgen Y. (2020). Characterization of carotid endothelial cell proliferation on Au, Au/GO, and Au/rGO surfaces by electrical impedance spectroscopy. Med. Biol. Eng. Comput..

[54] Tran A.K., Sapkota A., Wen J., Li J., Takei M. (2018). Linear relationship between cytoplasm resistance and hemoglobin in red blood cell hemolysis by electrical impedance spectroscopy & eight-parameter equivalent circuit. Biosens. Bioelectron..

[55] Yin X., Wu H., Jia J., Yang Y. (2018). A Micro EIT Sensor for Real-time and Non-destructive 3-D Cultivated Cell Imaging. IEEE Sens. J..

[56] Salehi B., Fokou P.V.T., Sharifi-Rad M., Zucca P., Pezzani R., Martins N., Sharifi-Rad J. (2019). The Therapeutic Potential of Naringenin: A Review of Clinical Trials. Pharmaceuticals.

[57] Goyal A., Verma A., Dubey N., Raghav J., Agrawal A. (2022). Naringenin: A prospective therapeutic agent for Alzheimer’s and Parkinson’s disease. J. Food Biochem..

[58] Sun T., Bernabini C., Morgan H. (2010). Single-colloidal particle impedance spectroscopy: Complete equivalent circuit analysis of polyelectrolyte microcapsules. Langmuir.

[59] Ahsan A.U., Sharma V.L., Wani A., Chopra M. (2020). Naringenin Upregulates AMPK-Mediated Autophagy to Rescue Neuronal Cells From β-Amyloid ((1-42)) Evoked Neurotoxicity. Mol. Neurobiol..

