# Supplementary material for: Amyloid-β Oligomer-Induced Electrophysiological Mechanisms and Electrical Impedance Changes in Neurons
Source: Sensors (Basel). 2024 Feb 14;24(4):1211. doi: 10.3390/s24041211 (PMC10892449; doi:10.3390/s24041211)
Supplement: Supplementary file 1 [file sensors-24-01211-s001.zip › sensors-2847813-supplementary.pdf]

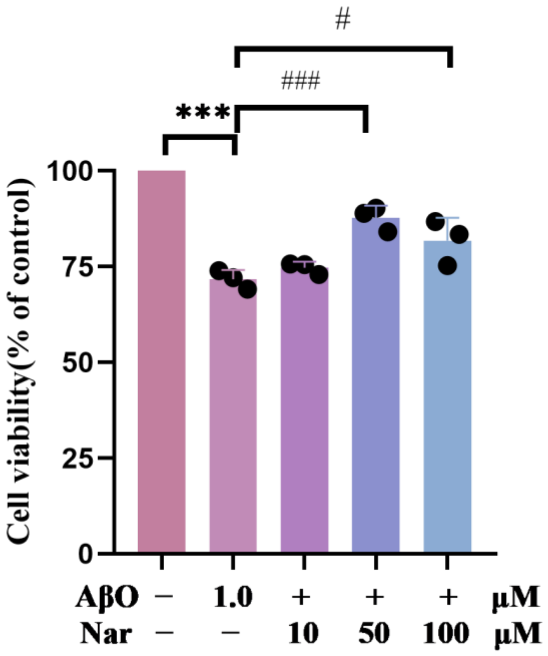

Figure S1. Cellular viability of AβOs and Nar on HT22 cells. Significance differences are indicated using "*" for the HT22 vs. HT22 + AβO groups and "#" for the HT22 + AβO vs. HT22 + AβO + Nar groups. [#] p < 0.05, ***/### p < 0.001. Three parallel experiments were performed as replications (n=3).
